# Association between physical measures of spinopelvic alignment and physical functioning with patient reported outcome measures (PROMs) after total hip arthroplasty: Protocol for systematic review and meta-analysis

**DOI:** 10.1371/journal.pone.0304382

**Published:** 2024-05-24

**Authors:** Sima Vatandoost, Katie Kowalski, Brent Lanting, K. C. Geoffrey Ng, Saghar Soltanabadi, Alison Rushton

**Affiliations:** 1 School of Physical Therapy, Western University, London, Ontario, Canada; 2 Department of Surgery, Division of Orthopaedic Surgery, Schulich School of Medicine and Dentistry, Western University, London, Ontario, Canada; 3 Department of Medical Biophysics, Schulich School of Medicine and Dentistry, Western University, London, Ontario, Canada; Public Library of Science, UNITED KINGDOM

## Abstract

**Introduction:**

Prevalence of total hip arthroplasty (THA) has trended upwards over past decades and is projected to increase further. Optimizing outcomes after surgery is essential to avoid surgical revision and maximize outcomes. Low back pain is reported as a problem post THA. Patient-reported outcome measures (PROMs) are commonly used to evaluate THA outcomes but have limitations (e.g., ceiling effects). It is therefore important to assess a comprehensive range of outcomes. Physical outcome measures of spinopelvic alignment and physical functioning demonstrate potential value, but no evidence synthesis has investigated their association with PROMs. The objectives of this systematic review are to evaluate the association between spinopelvic alignment and physical outcome measures of physical functioning with PROMs and characteristics of low back pain after THA.

**Methods and analysis:**

This protocol is aligned with the Preferred Reporting Items for Systematic Review and Meta-Analysis Protocols. Cross-sectional and longitudinal cohort studies evaluating the association between the physical outcome measures and PROMs (any outcome measures reported) following THA by any approach/implant will be included except surface replacement and revision THA. Studies investigating THA for developmental pathology and inflammatory conditions will be excluded. A systematic search in MEDLINE (Ovid), Embase (Ovid), Scopus, Web of Science, CINAHL, and the grey literature will be carried out from inception to July 31, 2023. Two independent reviewers will evaluate eligibility of retrieved articles, extract data and assess risk of bias (NIH quality assessment tool) of included studies. A third reviewer will mediate disagreements. Random-effects meta-analyses will be conducted if studies are sufficiently homogeneous in design, population, physical measures and PROMs; reporting odds ratios and 95% confidence intervals. Where meta-analyses are not possible, a narrative synthesis will be conducted. Confidence in cumulative evidence will be assessed using a modified GRADE (Grading of Recommendations Assessment, Development, and Evaluation).

**PROSPERO registration number:**

PROSPERO Registration number CRD42023412744.

## Introduction

Osteoarthritis (OA) is a leading cause of disability among older adults [1], with the hip joint commonly affected by this degenerative disease [2]. One of the most common and effective surgeries to alleviate pain and improve function in hip OA is total hip arthroplasty (THA) [3, 4]. Utilization of THA has followed an upward trend in the past two decades and is projected to continue to increase due to multiple factors including an aging population and increased life expectancy [5–7]. Optimizing outcomes after surgery can avoid surgical revision and poor clinical outcomes.

Patient-reported outcome measures (PROMs) are commonly used to evaluate outcomes following THA [8]. However, use of PROMs can be problematic owing to observed ceiling effects limiting their ability to evaluate higher levels of functional improvement [9, 10], and PROMs might not be correlated with physical outcome measures [11]. For example, although perceived physical functioning measured by PROMs was significantly improved four years following THA, Vissers et al found that other aspects of physical functioning like walking speed did not follow the same pattern [12]. Thus, physical outcome measures and PROMs may evaluate different constructs, making it essential to have a comprehensive assessment of multiple outcomes post THA. For THA, physical outcome measures of spinopelvic alignment and physical functioning are important to patient outcomes [13–15].

Spinopelvic alignment is defined as the interactions between the spine and pelvic regions, recognizing that a change in one region may lead to a reciprocal change in the other [16, 17]. In severe hip OA, patients can experience flexion contracture of the hip, and their spine is forwardly inclined with a higher risk of imbalanced alignment [18]. After THA surgery, radiographic parameters of spinopelvic alignment such as pelvic tilt and sacral slope may change, but the magnitude of these changes was not consistent amongst studies [19]. In addition, imbalanced spinopelvic alignment can be associated with poor clinical outcomes including function and quality of life. Spinopelvic alignment may also be associated with the presence of low back pain (LBP) after THA [13, 20, 21]. It has been observed that lumbar scoliosis and consequently LBP improved for patients who underwent unilateral THA [17]. However, it has also been observed by Eyvazov et al that LBP improvement was not associated with changes in spinopelvic alignment [22]. The evidence is therefore conflicting.

Physical functioning has been defined as the ability to do the physical activities of daily living [23], and as advised by OARSI, can be assessed using PROMs such as the Western Ontario and McMaster Universities Osteoarthritis Index (WOMAC) or physical outcome measures including the Timed Up & Go Test [24, 25]. An earlier systematic review had found a significant association between PROMs that evaluate preoperative physical function with functional outcomes following THA [26]. Although it has been reported that pain, quality of life and physical functioning assessed with PROMs commonly improve post THA [27], physical functioning assessed with physical outcome measures indicates remaining moderate-to-severe activity limitations for up to 30% of patients [28]. This suggests PROMs may not be comprehensive enough to assess physical functioning after THA [10, 29]. Furthermore, evidence demonstrates that LBP can impact THA outcomes, especially pain and function [30, 31]. However, the association of LBP on physical outcome measures of physical functioning has not been measured.

A recent scoping review has afforded further understanding of changes in spinopelvic alignment and LBP, demonstrating that both improved following THA [19]. No systematic review has investigated the association between physical measures of physical functioning and spinopelvic alignment with PROMs, and the association between these physical measures with LBP following THA.

### Objectives

The primary objective of this systematic review is to evaluate the association between spinopelvic alignment and physical measures of physical functioning with PROMs post THA. The secondary objective is to evaluate the association between the measures and LBP.

## Materials and methods

### Design

This protocol is written using the Preferred Reporting Items for Systematic Review and Meta-Analysis Protocols (S1 File) [32] and is registered in PROSPERO (CRD42023412744). The methodology is aligned with guidelines in the Cochrane Handbook [33].

### Eligibility criteria

#### Population

Participants following THA with no limitation on age will be included. Surgery due to developmental pathology categorized as a high level of dysplasia [34], inflammatory disorders, infection, avascular necrosis, trauma, tumor in the hip joint, surface replacement arthroplasty, and revision THA will be excluded.

#### Intervention

THA by any approach and any implant will be included. THA is defined as the removal of the femoral head and neck, acetabular cartilage and labrum, and subchondral bone which are replaced by prosthesis [35]. The scoping search completed in preparing this protocol suggested there are a limited number of papers which investigate the associations in our objectives for the total hip arthroplasty population. This led to our decision to not to put any limitations on the site, type of THA and implant being used, the condition of the other hip, and etiology of hip arthritis. This decision will ensure that we have enough studies to include in this review.

#### Comparator

No comparator is required for this review.

#### Outcome measures

The measures include spinopelvic alignment, physical outcome measures of physical functioning, and PROMs as defined below.

Spinopelvic alignment—including any parameters from the spine to the pelvis that contribute to maintaining an energy-efficient posture in normal or pathological status (e.g., thoracic kyphosis, lumbar lordosis, pelvic tilt, acetabular inclination) [19, 36].

Impairment-based physical outcome measures—evaluating abnormal structure or dysfunction in the specific body part or system (e.g., muscle strength, muscle endurance, functional muscle performance such as chair stand test) [37].

Performance-based physical measures—assessing performance on a specific task in a standardized manner (e.g., 6-minute walk test) [37, 38].

Physical outcome measures that evaluate activity in the natural environment (e.g., accelerometry) [38].

PROMs—defined as any outcome measure directly reported by patients [39]. We put no limitation on the type of PROMs.

LBP—defined by pain and discomfort in the regions below the costal margin and above the inferior gluteal folds, characterized with or without leg pain [40] with no limitation on the type of LBP.

#### Study design

Studies that evaluate the association between spinopelvic alignment and/or physical measures of physical functioning with PROMs will be included. Observational studies including retrospective and prospective longitudinal cohorts and cross-sectional studies that evaluate associations after surgery will be included. We put no limitations on the language of the studies.

### Information sources

A systematic search in the following electronic databases will be conducted from inception to July 31, 2023: MEDLINE (Ovid), Embase (Ovid), Scopus, Web of Science and CINAHL. The grey literature will be searched in Dissertations and Theses of ProQuest (https://www.proquest.com/) and conference proceedings/abstracts in Web of Science, and Scopus. The reference lists of included studies and the table of contents in the *Journal of Arthroplasty*, *the Journal of Bone & Joint Surgery—American Volume* (high volume of THA publications) will be reviewed to ensure saturation of all eligible studies.

### Search strategy

The search strategy (S2 File) has been developed by the first author with the assistance of coauthors and an experienced librarian. The search has been developed in MEDLINE (Ovid) based on the medical subject headings and free text words for population, intervention, study design, and outcome measures, and then the alternative MESH terms for each parameter have been added by putting OR within each group of concepts [33]. There is no restriction on the language of studies to ensure that we identify all relevant studies in all languages. In the final stage, the four groups of keywords have been combined using the AND operator [33]. The keywords and syntax will be modified to be applied in other databases. Free text words for hip OA, THA, and LBP were selected from the most relevant guidelines and systematic reviews [41–43]. Search terms and outcome measures mentioned in the recent scoping review were used to define the free text words for spinopelvic alignment [19]. Regarding physical outcome measures of physical functioning, keywords were defined based on a recent systematic review protocol [44]. The text words for the main constructs of PROMs were selected from the Patient-Reported Outcomes Measurement Information System (PROMIS) [45]. Based on our scoping search, as pain, physical functioning, disability, and quality of life are commonly measured for THA [46–48], all the commonly used questionnaires that evaluate these outcomes were included as search terms (e.g., Visual Analogue Scale, Oswestry Disability Index, 36-Item Short Form Survey (SF-36)). Due to the difficulty in the terminology of the physical functioning patient-reported outcome measures, we include all the list of questionnaires presented by The Initiative on Methods, Measurement, and Pain Assessment in Clinical Trials (IMMPACT) and Outcome Measures in Rheumatology (OMERACT) to have a comprehensive search [38]. Finally, the synonyms for the free text words of all categories were defined to keep the search strategy as broad as possible and capture all the relevant studies.

### Study records

#### Data management

Citations will be imported and sorted in Covidence, a web-based software tool for conducting systematic reviews [49]. The software will identify duplicates and remove them automatically. When the title and abstract have been screened, the full text of eligible studies will be uploaded and stored in Covidence.

#### Selection process

**Fig 1:**
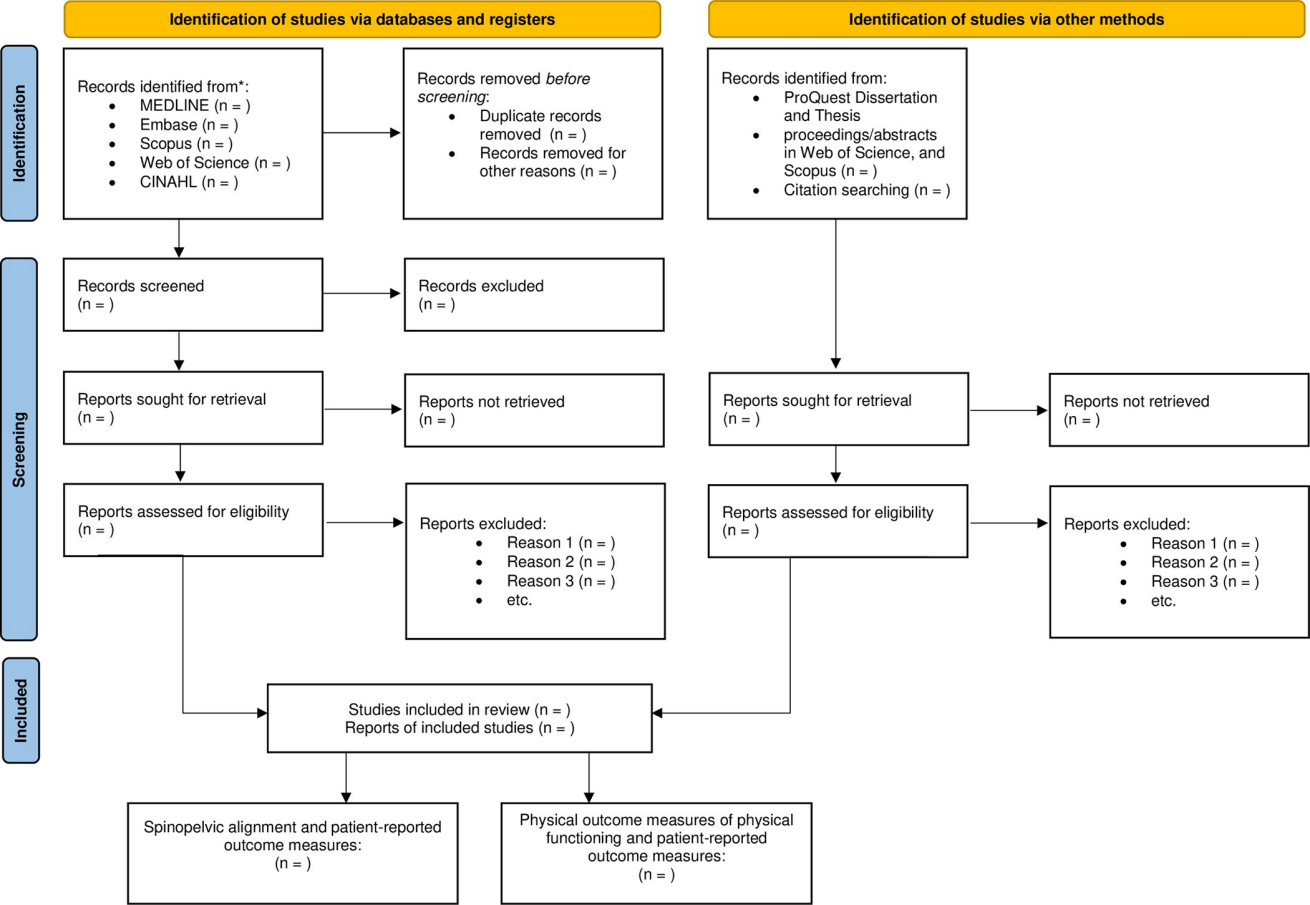
Flow diagram of study selection processes.

Eligibility criteria will be evaluated by two independent reviewers (SV and SS) in two stages. The title and abstract will be initially screened. Then, the full text of studies that are potentially eligible or do not show sufficient information in the title and abstract to determine eligibility will be obtained. The reviewers will screen the full text independently for determination of inclusion. To translate the Non-English studies selected for the full-text screening, we will use a three-stage process. We will first contact the corresponding author through email to ask for the English version of the study. If an English version of the study is not available, we will translate the study into English through Google Translate and then request that the corresponding author proof-read the translated version to ensure the accuracy of the translation. If we do not hear it back from them, a follow-up email will be sent after two weeks. In case the author does not respond, we will contact the International and Exchange Student Centre at Western University to link us with native students in that language to request their proof reading expertise. We will acknowledge that the translated version of these studies has been used in the manuscript to ensure full transparency. Any disagreements will be discussed, and if both reviewers agree the eligibility criteria have been met, the study will be included. When they fail to reach consensus in either stage, the third reviewer (ABR) will make the final decision. Cohen’s kappa will be used to evaluate agreement between reviewers in both stages [50]. All stages of this process and reasons why studies are excluded will be summarized in the Preferred Reporting Items for Systematic Reviews and Meta-Analyses flow diagram (Fig 1) [51].

#### Data collection process

Two reviewers (SV and SS) will independently perform data extraction from included studies by using standardized data collection forms. The forms will be piloted with five articles and modified if required to ensure they collect information aligned with the purpose of the review. Any disagreements in the data extracted will be discussed and resolved between reviewers. If not resolved, the third reviewer (ABR) will mediate. If data are unclear or missing in the included studies, the corresponding author will be contacted via email for further information. Where the author does not respond, two follow-up reminder emails will be sent at two-week intervals. If the authors do not address the request about incomplete data, we will report that the data is incomplete and explain the parts at which enough data was not provided.

### Data items

Data items to be extracted from the eligible studies are shown in Table 1.

**Table 1:** Summary of data to be extracted from included studies.

Study characteristics	Authors, year of publication, study design, country of study, language, objective(s) of the study, source of funding
Study participants	Age, sex, number of included participants, arthroplasty approach (anterior, lateral, posterior), type of arthroplasty (bilateral or unilateral, conventional or dual mobility THA), history of LBP, and other health conditions
Outcome measures	Type, equipment required, number of assessments (before and after the surgery), type of statistical measure, results

### Risk of bias in individual studies

The NIH quality assessment tool for observational cohort and cross-sectional studies will be used to evaluate the risk of bias (internal validity) in the included studies [52] by two independent reviewers (SV and SS). In case of disagreements, they will discuss to reach a consensus, and if they fail to resolve the conflicting views, a third reviewer (ABR) will be asked to mediate. Cohen’s kappa will be used to assess the agreement between reviewers [50]. The NIH is a quality assessment tool that is valid and reliable for observational cohort and cross-sectional studies [52]. It comprises fourteen questions which are rated as: "yes, no, and cannot determine" and will be rated as poor, fair, or good if the study meets 0–4, 5–10, and 11–14 questions that were answered yes out of 14 questions, respectively [53].

### Data synthesis

If studies are sufficiently homogeneous based on design, population as a whole (primary objective) and those who experience LBP (secondary objective), outcome measures and analyses to pool data for statistical analysis, meta-analyses will be conducted. The postoperative timeline of the studies will be presented for short-term (≤ 3 months), medium-term (> 3, ≤ 12 months), and long-term (> 12 months) follow-up [54]. Odds ratios obtained through a random-effects model with 95% confidence intervals will be used [55]. Heterogeneity will be assessed through the I^2^ test [55, 56]. Where meta-analyses cannot be conducted due to heterogeneity, a narrative synthesis will be conducted based on the Cochrane Consumers and Communication Review Group: Data synthesis and analysis guideline [57], excluding the first step of "developing a theory of how the intervention works" because this review aims to evaluate association rather than effectiveness of an intervention. The narrative synthesis will be structured around the study design, risk of bias of included studies, population categories including with and without LBP, type of outcome measures and statistical parameters [57]. A detailed overview of the "overall completeness and applicability of evidence" together with methodological issues or potential biases will be presented [57].

### Subgroup analysis

The scoping search completed in preparing this protocol suggested it is not possible to have a subgroup analysis because there were a limited number of papers on this topic, and heterogeneity was clear among them.

### Meta-bias (es)

Reporting bias will be assessed through a search for protocols of included studies and the assessment of the consistency between the study protocols and published results.

### Confidence in cumulative evidence

Grading of Recommendations Assessment, Development, and Evaluation (GRADE) will be used to rate the overall quality of evidence based on the criteria including risk of bias, inconsistency, imprecision, indirectness, and publication bias. GRADE will be adapted for this review, based on the assessment of cumulative evidence for prognostic factors that evaluates association [58, 59]. In this review, indirectness will address whether the population and outcome measures of included studies correspond to our population and outcome of interest. Certainty will be rated based on the effect estimates that differ from a relative effect of 1.0. In the case of conducting a meta-analysis, the author will evaluate inconsistency by the I^2^ test and differences in effect size by taking overlap in confidence intervals into consideration. This approach will rate the evidence as high, moderate, low, and very low [58]. GRADE will be assessed by two independent reviewers (S.V. and S.S.) and any disagreements will be solved through discussion. When consensus cannot be achieved, the third reviewer (ABR) will mediate.

### Patient and public involvement

The development of the protocol has been discussed with the spinal pain research Patient Partner Advisory Group in the School of Physical Therapy at Western University. They will also be informed about the findings of this review. The discussion aims to obtain their feedback on the process of conducting the review and the interpretation of the results.

## Discussion

Preoperative planning is critical to perform successful THA surgery [60]. One way to achieve this goal is to have a clear understanding of spinopelvic parameters and how they are associated with clinical outcomes post-surgery [13, 60, 61]. In addition, physical measures of physical functioning and their association with PROMs can play a critical role in understanding limitations after surgery [29]. Given the importance of evaluating multidimensional outcomes post-surgery, this review will help us to understand the relationship between physical measures with clinical outcomes.

Physical measures may also be associated with LBP [13, 20, 21], which has a prevalence of 21.2% to 49.4% in patients with hip OA [62] and can affect the clinical outcome after THA. Although patients with hip OA tend to have abnormal spine-hip relations or limited physical function [63, 64], it has not been clarified how changes in these measures can affect LBP following THA. At the end of this systematic review, we will have insight into the physical measures associated with LBP among this population as a starting point for further research.

Additionally, the review will inform healthcare professionals and researchers about outcome measures that may be valuable to measure post-surgery. This will enable further research to improve patient examination and targeting of interventions pre and/or post-surgery to optimize outcomes following THA.

## Supporting information

S1 File PRISMA-P checklist. (DOC)

S2 File Search strategy in the MEDLINE Ovid. (DOCX)
