# Supplementary material for: Association between physical measures of spinopelvic alignment and physical functioning with patient reported outcome measures (PROMs) after total hip arthroplasty: Protocol for systematic review and meta-analysis
Source: PLoS One. 2024 May 24;19(5):e0304382. doi: 10.1371/journal.pone.0304382 (PMC11125517; doi:10.1371/journal.pone.0304382)
Supplement: S2 File — (DOCX) [file pone.0304382.s002.docx]

**Example of search strategy developed in MEDLINE (Ovid)**

1 (hip adj4 (osteoarthriti* or arthriti* or arthros* or osteoarthrosi*)).tw,kf.

2 Hip Joint/ or Osteoarthritis, Hip/ or Arthroplasty, Replacement, Hip/ or Hip Prosthesis/ or Hip/

3 (hip joint prosthes* or hip prosthes* or THA).tw,kf.

4 (hip adj4 (remov* or surg* or replac* or arthroplast*)).tw,kf.

5 coxarthrosi*.tw,kf.

6 (hip implant* or artificial hip joint*).tw,kf.

7 or/1-6

8 (spin* align* or vertebra* align* or spin* column or vertebra* column or spinopelvic align* or lumbosacral align* or sagittal align*).tw,kf.

9 (spine-hip relation* or cervic* vertebra* or thoracic* align* or lumbar* align* or sacral* align* or hip* angle* or spinopelvic* or alignment*).tw,kf.

10 ((pelvis* or pelvic* or hip* or spin*) adj4 alignment).tw,kf.

11 cervical lordos*.tw,kf.

12 thoracic kyphos*.tw,kf.

13 lumbar lordos*.tw,kf.

14 lumbar scolios*.tw,kf.

15 T1 spinopelvic inclination*.tw,kf.

16 pelvic tilt*.tw,kf.

17 T1 pelvic*.tw,kf.

18 sacral slope*.tw,kf.

19 pelvic incidence*.tw,kf.

20 pelvic inclination*.tw,kf.

21 anterior pelvic plane*.tw,kf.

22 sagittal vertical axis*.tw,kf.

23 spinosacral*.tw,kf.

24 (cup inclination* or acetabular inclination*).tw,kf.

25 cup anteversion*.tw,kf.

26 acetabular anteversion*.tw,kf.

27 or/8-26

28 (physical outcome* or physical* measure* or physical* assess* or objective* outcome* or objective* assess* or objective* measure* or physical function* or function* measure*).tw,kf.

29 "Range of Motion, Articular"/

30 (range of motion or ROM).tw,kf.

31 Schober.tw,kf.

32 (finger* adj4 (floor or toe or knee or fibular head)).tw,kf.

33 (wrist crease adj3 floor).tw,kf.

34 (straight leg raise* or SLR or Lasegue*).tw,kf.

35 (isometric strength or isokinetic strength).tw,kf.

36 (motor control or movement control or functional movement screen* or FMS or sitting one leg knee extension or posterior pelvic tilt or waiter's bow or one leg stance).tw,kf.

37 gait/ or gait analysis/

38 walking speed/

39 (spatiotemporal gait or spatio temporal gait or stride length or stride duration or gait speed or cadence or gait asymmetry or stance phase or swing phase or double limb support or single limb support).tw,kf.

40 (inclinometer or goniometer or kyphometer or electromagnetic tracking).tw,kf.

41 muscle strength dynamometer/

42 (dynamometer or manual muscle test or MedX or Cybex or Kin-Com or RehaGait or JAMAR).tw,kf.

43 (aerobic capacity or VO2* or bicycle ergomet* or maximal graded exercise* or steep ramp).tw,kf.

44 (Biering Sorensen or modified Sorensen).tw,kf.

45 Roman chair.tw,kf.

46 (sternum adj3 (ground or floor)).tw,kf.

47 muscle endurance.tw,kf.

48 ((back or body or trunk) adj3 endurance).tw,kf.

49 prone bridge.tw,kf.

50 ((lower extremities or (hips and knees)) adj5 (90deg* or "90 degree*")).tw,kf.

51 (arch-up* or sit-up* or squat* or dumbbell press*).tw,kf.

52 (double limb stance or single limb stance or stork stand* or flamingo balance or y-balance).tw,kf.

53 CTSIB.tw,kf.

54 (clinical test* adj2 sensory interaction adj2 balance).tw,kf.

55 (clinical test* adj2 sensory integration adj2 balance).tw,kf.

56 (Berg balance scale or Tinetti* or performance oriented mobility assessment* or tandem walk*).tw,kf.

57 lower extremity motor coordination test*.tw,kf.

58 (chair adj3 (stand* or rise*)).tw,kf.

59 (sit to stand or stand up or stand ups or roll*).tw,kf.

60 (lie adj2 sit).tw,kf.

61 (bed adj2 chair).tw,kf.

62 step*.tw,kf.

63 ((stand or standing) adj2 continuous).tw,kf.

64 functional capacity evaluation.tw,kf.

65 lifting/

66 functional reach.tw,kf.

67 (lift* or progressive isoinertial lifting evaluation or pile).tw,kf.

68 forward reach.tw,kf.

69 walk test/

70 (self-paced walk* or 4-meter walk* or 4-metre walk* or 5-meter walk* or 5-metre walk* or 10-meter walk* or 10-metre walk* or 15-meter walk* or 15-metre walk* or 50-meter walk* or 50-metre walk* or 50-foot walk* or 5-minute walk* or 6-minute walk* or treadmill or overground walk*).tw,kf.

71 (walk adj3 hall*).tw,kf.

72 shuttle walk*.tw,kf.

73 (stair* adj2 climb*).tw,kf.

74 ("timed up and go" or TUG or "8 foot up and go").tw,kf.

75 (Physical capability assessment tool or PCAT or aggregated functional performance test or aggregated assessment of physical function or short physical performance battery or cumulated ambulation score or functional independence measure or Katz ADL index).tw,kf.

76 (Activity measure for post-acute care 6 clicks or Activity measure for postacute care 6 clicks).tw,kf.

77 (Physiotherapy functional mobility profile or Barthel index).tw,kf.

78 lying.tw,kf.

79 (time adj5 stand*).tw,kf.

80 (constant postures or active postures or sedentary postures or sedentary activity or walking time or walking distance or claudication index or walking speed or daily walking events or light intensity or moderate intensity or vigorous intensity or activity count or gait cycles or gait posture index or physical activity).tw,kf.

81 accelerometry/

82 wearable electronic devices/ or fitness trackers/

83 (acceleromet* or activity monitor or pedomet* or GPS or watch or smartwatch).tw,kf.

84 (6WT adj3 app*).tw,kf.

85 (muscle function or muscle length or muscle strength or muscle activity or EMG activity).tw,kf.

86 (muscle activ* or electromyography activ* or muscle stength test* or muscle strength grad* or muscular* assess*).tw,kf.

87 or/28-86

88 27 or 87

89 Pain Measurement/

90 Disability Evaluation/ or "International Classification of Functioning, Disability and Health"/

91 Patient Satisfaction/ or Motivation/ or Psychological/ or Personal Satisfaction/ or Self Efficacy/ or Self Care/

92 Interpersonal Relations/ or Social Behavior/ or Social Participation/ or Social Support/ or Social Isolation/

93 "Quality of Life"/ or Anxiety/ or Depression/ or Emotions/ or Stress, Psychological/ or Anger/ or Cognition/

94 Kinesiophobia/

95 "Surveys and Questionnaires"/

96 Patient expect*.tw,kf.

97 Visual Analogue Scale.tw,kf.

98 Physical functioning patient reported outcome*.tw,kf.

99 Treatment Outcome/ or Patient Reported Outcome Measures/ or Patient Outcome Assessment/

100 Back Pain/ or Low Back Pain/

101 (subjective* outcomes or subjectively measured outcome* or subjective assessment of outcome* or patient reported* or subjective* measure or PROM*).tw,kf.

102 ((health-related quality of life or HRQOL) adj2 questionnaire).tw,kf.

103 ((Roland-Morris Disability or RMD) adj2 questionnaire).tw,kf.

104 (Low* back* pain* or low* back* ache* or backache* or lumbar vertebra* pain or lumbar spin* pain or lumbosacral pain or lumbo-sacral pain or lumbar* pain or lumbago* or sciatica or radiculopath* or radicular pain*).tw,kf.

105 Oxford Hip Score.tw,kf.

106 harris hip score.tw,kf.

107 EQ-5D.tw,kf.

108 SF-36*.tw,kf.

109 ((hip disability and osteoarthritis outcome*) or HOOS*).tw,kf.

110 ((Western Ontario and McMaster Universities Arthritis Index) or WOMAC).tw,kf.

111 (Numeric* Rating Scale or NRS).tw,kf.

112 McGill Pain Questionnaire.tw,kf.

113 Tampa Scale of Kinesiophobia.tw,kf.

114 Beck Depression Inventory.tw,kf.

115 Nottingham Health Profile.tw,kf.

116 fear-avoidance beliefs questionnaire.tw,kf.

117 UCLA loneliness scale.tw,kf.

118 (Japanese Orthopaedic Association Hip-Disease Evaluation Questionnaire or JHEQ).tw,kf.

119 HOOS JR.tw,kf.

120 (Forgotten Joint Score-12 or FJS-12).tw,kf.

121 (short questionnaire to assess health-enhancing physical activity or SQUASH*).tw,kf.

122 (The Veterans RAND 12-Item Health Survey or VR-12*).tw,kf.

123 Likert scale.tw,kf.

124 ((The Copenhagen Hip and Groin Outcome Score) or HAGOS*).tw,kf.

125 ((The pain and function of the hip) or PFH*).tw,kf.

126 (the postoperative recovery profile or PRP*).tw,kf.

127 The Pain Disability Questionnaire.tw,kf.

128 Chronic Pain Self Efficacy Scale.tw,kf.

129 Pain Self-Efficacy Questionnaire.tw,kf.

130 The Pain Disability Index.tw,kf.

131 (Questionnaire for Physical Activity Decline in Pain or PAD).tw,kf.

132 Daily Activity Diary for Chronic Pain Patients.tw,kf.

133 the Multidimensional Pain Inventory.tw,kf.

134 the Brief Pain Inventory.tw,kf.

135 ((The Impact on Participation and Autonomy) or IPA).tw,kf.

136 ((The Impact on Participation and Autonomy Questionnaire) or IPAQ).tw,kf.

137 The Physical Activity Questionnaire.tw,kf.

138 (The Quality of Well-Being Scale or QWB).tw,kf.

139 (The Sickness Impact Profile or SIP).tw,kf.

140 (Work Limitations Questionnaire or WLQ).tw,kf.

141 Human Activity Profile.tw,kf.

142 Motor Fitness Scale.tw,kf.

143 PROMIS.tw,kf.

144 (Population Surveys of Chronic Disease and Disability).tw,kf.

145 the CDC HRQOL-14.tw,kf.

146 The Duke-UNC Health Profile.tw,kf.

147 Oswestry Disability Index.tw,kf.

148 Health Assessment Questionnaire.tw,kf.

149 Functional Status Questionnaire.tw,kf.

150 Rosow Breslau Index of Mobility.tw,kf.

151 Short Musculoskeletal Function Assessment Questionnaire.tw,kf.

152 Musculoskeletal Functional Limitation Index.tw,kf.

153 Patient-specific activity scoring scheme.tw,kf.

154 or/89-153

155 (observational adj5 (study or studies or design or analysis or analyses)).ti,ab,kf.

156 (prospective adj5 (study or studies or design or analysis or analyses)).ti,ab,kf.

157 (retrospective adj6 (study or studies or design or analysis or analyses or data)).ti,ab,kf.

158 ((longitudinal or longterm or long term) adj6 (study or studies or design or analysis or analyses or data)).ti,ab,kf.

159 (cross sectional adj8 (study or studies or design or research or analysis or analyses or survey or findings)).ti,ab,kf.

160 (associat* or regression* or correlat* or relat*).tw,kf.

161 cohort*.ti,ab,kf.

162 Cohort Studies/

163 Observational Study/

164 Cross-Sectional Studies/

165 Prospective Studies/

166 Retrospective Studies/

167 Longitudinal Studies/

168 or/155-167

169 7 and 88 and 154 and 168
